# Should police have access to genetic genealogy databases? Capturing the Golden State Killer and other criminals using a controversial new forensic technique

**DOI:** 10.1371/journal.pbio.2006906

**Published:** 2018-10-02

**Authors:** Christi J. Guerrini, Jill O. Robinson, Devan Petersen, Amy L. McGuire

**Affiliations:** Center for Medical Ethics and Health Policy, Baylor College of Medicine, Houston, Texas, United States of America

## Abstract

On April 24, 2018, a suspect in California’s notorious Golden State Killer cases was arrested after decades of eluding the police. Using a novel forensic approach, investigators identified the suspect by first identifying his relatives using a free, online genetic database populated by individuals researching their family trees. In the wake of the case, media outlets reported privacy concerns with police access to personal genetic data generated by or shared with genealogy services. Recent data from 1,587 survey respondents, however, provide preliminary reason to question whether such concerns have been overstated. Still, limitations on police access to genetic genealogy databases in particular may be desirable for reasons other than current public demand for them.

## Introduction

On April 24, 2018, a suspect in California’s notorious Golden State Killer cases was arrested after decades of eluding the police. The capture of Joseph James DeAngelo, a former police officer, was a critical step toward closing the books on 12 unsolved murders and at least 45 rapes that were committed throughout California from 1976 to 1986 [1]. While DeAngelo’s arrest was widely celebrated, concerns linger regarding the forensic techniques that ultimately brought him to justice. That is because the police identified DeAngelo by first identifying his relatives using a free, online genetic database populated by individuals researching their family trees. By participating in genetic genealogy databases and using other personal genetic services intended to facilitate self-discovery, individuals can become criminal informants vis-à-vis their own families. But should this be allowed?

## Shrinking the haystack

For decades, California police tracked the Golden State Killer using traditional detective work, but all leads came to dead ends. Six years ago, however, investigators tried a different tactic. Instead of trying to identify the killer—a needle in an enormous haystack—they sought first to reduce the size of the haystack by identifying the killer’s family. Investigators did so by uploading a genetic profile generated from DNA that had been collected from one of the crime scenes to genealogy websites that match users to their genetic relatives [1].

After years of searching and at least one failed attempt [2], investigators finally identified the extended family of the Golden State Killer using the website GEDmatch. GEDmatch, which is operated by a handful of genealogy hobbyists, compares users’ genetic data (originally obtained from direct-to-consumer [DTC] genetic testing services like 23andMe and AncestryDNA) to identify genetic matches among them. The website’s algorithm generated a partial match between the killer’s DNA, which investigators submitted under a fake name, and the DNA of at least one distant relative [3].

Having substantially reduced the haystack, investigators were able to identify the needle by constructing family trees and scouring them for potential suspects. Investigators eventually zeroed in on DeAngelo and matched his genetic data, which was collected from an object that he discarded while under surveillance, to crime scene DNA [1].

## Probing public opinion

DeAngelo’s arrest was widely celebrated as a clever investigative coup that may have finally brought to justice a man who had terrorized California residents for years. Yet as details of his arrest emerged, privacy concerns were quickly raised about police searches of personal genetic data of the kind used to capture DeAngelo. Law enforcement officers routinely query the “offender database” of the Federal Bureau of Investigation (FBI)-maintained Combined DNA Index System (CODIS), which holds the genetic profiles of known felons, misdemeanants, and arrestees, for matches to crime scene data [4]. But in the manhunt for the Golden State Killer, officers queried the genetic data of individuals who had done nothing to raise police suspicions.

If there ever was any question, it is now clear that these data are vulnerable to police access—and are increasingly so as investigators turn to them to solve other crimes. Efforts to apply the same technique that identified DeAngelo to other criminals are already underway and have helped identify suspects in at least four other cold murder cases and one recent rape case [5, 6]. Meanwhile, Parabon NanoLabs has partnered with law enforcement to conduct familial genetic searches for criminals and has uploaded more than 140 profiles to GEDmatch since May 2018 [7].

Some have suggested that individuals who contribute to genetic genealogy databases have an expectation of privacy in their genetic data that may be violated by familial genetic searches [8]. Although many genetic service providers warn users about the potential for third parties, including law enforcement, to access personal genetic data, these warnings are usually buried in privacy policies and terms of service that users may never read. Yet among those who do appreciate these potential forensic uses, not all may be particularly disturbed by them [8, 9].

To date, public opinion on this issue has not been assessed, although it is a critical input to policy discussions regarding whether police should be permitted to access data held by personal genetic service providers, including but not limited to searching genetic genealogy databases for the purpose of generating investigative leads. To begin filling this knowledge gap, in May 2018, we distributed a 20-item survey to assess individual perspectives on police access to genetic genealogy websites and customer information from DTC genetic testing companies. We conducted the survey online using Amazon Mechanical Turk (MTurk), a well-known recruiter for survey research reported in scientific journals [10, 11]. We restricted participation to individuals who were 18 years of age or older and located in the United States and paid them US$0.25 for taking the survey. The methods, survey, and survey data are set forth in supporting information files (S1 Text, Methods and data analysis; S2 Text, Survey; and S1 Data, Raw survey data).

**Fig 1:**
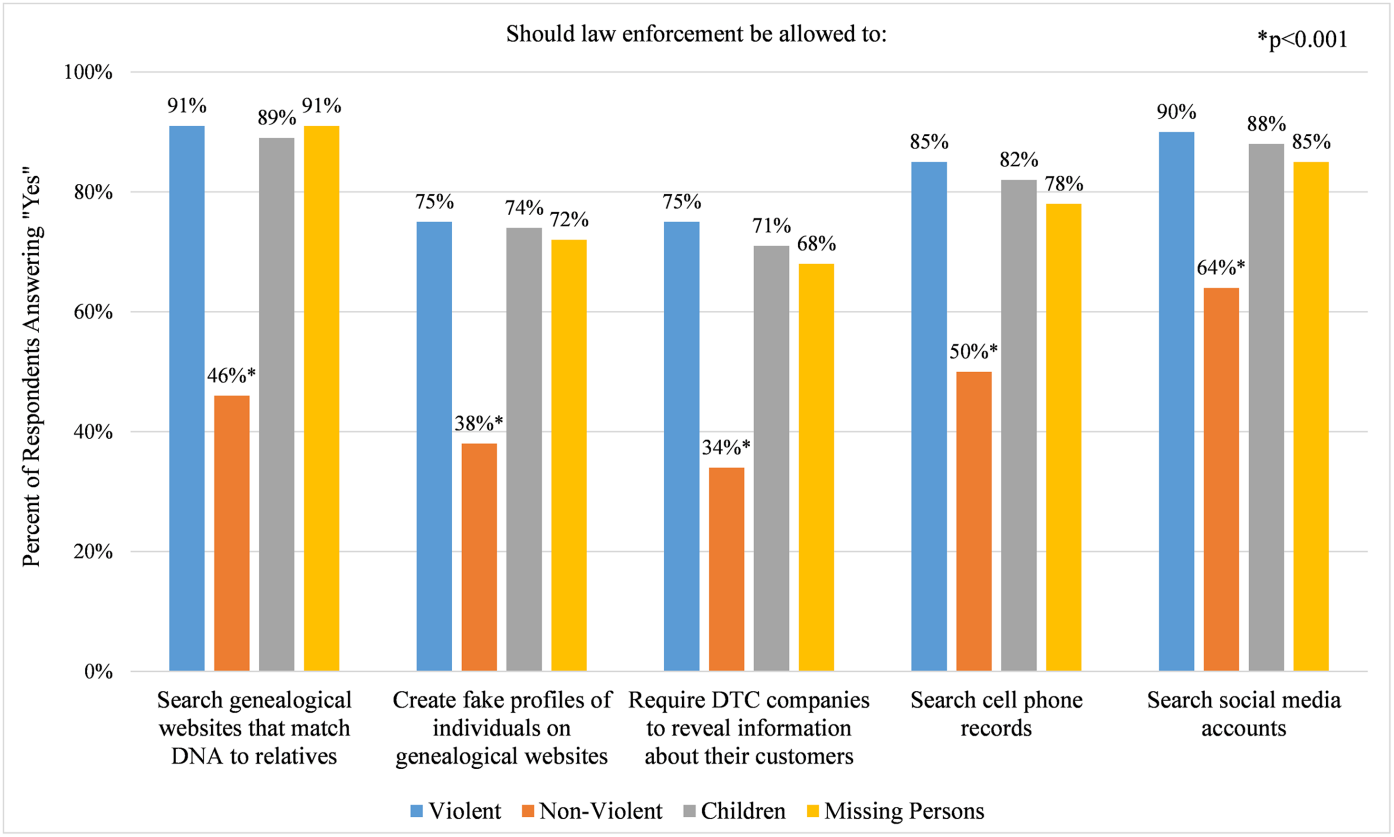
Percent of respondents favoring police access to information for specific investigative purposes. Investigative purposes are categorized as (a) Violent = to identify perpetrators of violent crimes (for example, rape, murder, arson, or kidnapping); (b) Non-Violent = to identify perpetrators of nonviolent crimes (for example, car theft or drug possession); (c) Children = to identify perpetrators of crimes against children (for example, child abuse); and (d) Missing Persons = to identify missing persons.

Among the 1,587 respondents (Table 1), the majority supported police searches of genetic websites that identify genetic relatives (79%) and disclosure of DTC genetic testing customer information to police (62%), as well as the creation of fake profiles of individuals by police on genealogy websites (65%) (Fig 1). However, respondents were significantly more supportive of these activities (all *p* < 0.05) when the purpose is to identify perpetrators of violent crimes (80%), perpetrators of crimes against children (78%), or missing persons (77%) than when the purpose is to identify perpetrators of nonviolent crimes (39%). Notably, a similar line was drawn by GEDmatch in its updated privacy policy, adopted after the survey was closed, which explicitly permits law enforcement to search GEDmatch for matches to DNA left at scenes of violent crimes, defined by the site as homicide and sexual assault [12].

**Table 1:** Respondent characteristics.

Characteristic–*N* (%) unless otherwise noted	N = 1,587
*Age*	
18–22	121 (7.6%)
23–36	880 (55.5%)
37 or older	586 (36.9%)
Gender*	
Male	761 (48.2%)
Female	818 (51.8%)
*Race/ethnicity*	
Non-Hispanic white	1,130 (71.2%)
Other^+^	457 (28.8%)
*Annual household income*	
≤ US$49,999	822 (51.8%)
≥ US$50,000	765 (48.2%)
*Respondent/family member victim of a crime*	
No	648 (40.8%)
Yes	939 (59.2%)
*Respondent/family member arrested for a crime*	
No	1,013 (63.8%)
Yes	574 (36.2%)
*Respondent/family member convicted of a crime*	
No	1,125 (70.9%)
Yes	462 (29.1%)
*Respondent/family member held a law enforcement job*	
No	1,207 (76.1%)
Yes	380 (23.9%)
*Purchased DTC genetic testing*	
No	1,398 (88.1%)
Yes	189 (11.9%)
*Researched relatives on genealogy website*	
No	1,002 (63.1%)
Yes	585 (36.9%)

* Gender does not sum to total N due to participant nonresponse.

^+^ Other includes Hispanic/Latino, American Indian or Alaska Native, Black or African American, Asian, and Other.

**Abbreviation:** DTC, direct-to-consumer.

Females were significantly more likely to support these police activities than males (β = 0.051, *p* < 0.001). However, age; race and ethnicity; annual household income; purchase of DTC genetic testing services; use of genealogy websites to research relatives; personal or relative’s victimization, arrest, or criminal conviction; and personal or relative’s employment in law enforcement were not independent predictors of support in a multivariate model (all *p* > 0.05) (Table 2).

**Table 2:** Participant characteristics predictive of support for police activities to solve violent crimes.

Characteristic	Unstandardized β	Standardized β	*p-*value	95% confidence interval
*Age*	−0.011	0.030	0.727	−0.70–0.049
*Gender*	0.240	0.051	<0.001	0.140–0.341
*Race/ethnicity*	0.071	0.056	0.206	−0.039–0.180
*Annual household income*	0.016	0.015	0.270	−0.012–0.045
*Respondent/family member victim of a crime*	0.090	0.055	0.103	−0.018–0.198
*Respondent/family member arrested for a crime*	−0.011	0.086	0.894	−0.179–0.156
*Respondent/family member convicted of a crime*	0.098	0.089	0.270	−0.076–0.272
*Respondent/family member held a law enforcement job*	−0.037	0.060	0.535	−0.155–0.080
*Purchased DTC genetic testing*	−0.027	0.082	0.742	−0.187–0.133
*Researched relatives on genealogy website*	0.010	0.005	0.857	−0.100–0.120

For comparison, we also assessed respondents’ perspectives on police access to cell phone records and social media accounts in the possession of communications service providers. Warrantless seizures and searches of historical cell phone location information was at issue in *Carpenter versus US*, which was pending before the US Supreme Court when the survey was conducted [13]. We found exactly the same pattern of strong support for police access to cell phone records and social media accounts except when the purpose is to identify perpetrators of nonviolent crimes (Fig 1).

## Developing policy parameters

These data provide preliminary evidence that individuals may not be particularly concerned about police searches of personal genetic data that populate genetic genealogy databases when the purpose is considered justified. Notably, although arrestees and convicted criminals are disproportionately low income and black [14] and the prototypical customer of DTC genetic testing services is high income and white [15], such disparities did not translate into differences in respondents’ support based on income or race. If this finding proves consistent across subsequent surveys and among more diverse populations, it has policy implications to the extent that forensic use of genetic genealogy databases might serve to balance forensic use of CODIS, which reflects the racial disparities of the criminal justice system [16]. It may be that the individuals who will provide that balance do not object to doing so.

Although MTurk samples have been shown to be more diverse than convenience samples [17], the respondent population may have differed from the general population in several respects. For one, compared to national statistics collected on adults by the US Census Bureau, our survey respondents were younger: the median age category of our participants was 23–36 years old versus 37 years nationally [18]. In another MTurk survey, some of us found that concerns about privacy and security of online and health information spanned all ages, although younger generations reported being significantly less likely to have such concerns than older generations [11]. Notably, we did not find age to be a significant predictor of support for police access in our multivariate analysis (Table 2).

In addition, the majority of respondents (59%) reported that they or family members had been victims of crimes. By comparison, in 2016, the National Crime Victimization Survey (NCVS) administered by the US Bureau of Justice Statistics found that 1.3% of persons aged 12 or older were victims of violent crimes and 8.8% of households were victims of property crimes [19]. However, unlike the NCVS, which produces annual estimates of victimization, our survey asked about incidents of victimization over one’s lifetime, and it also did not restrict family member victims to only those living in the same household. Thus, it is not surprising that our survey respondents reported a higher prevalence of victimization. Still, it is possible that because of their experiences, our survey respondents demonstrated a bias in favor of police access unlike that which would be found among the US general population.

Likewise, nearly a quarter of our survey respondents (24%) reported that they or family members had ever been employed in law enforcement, which was broadly defined to include security guards and bailiffs. As of May 2017, the US Department of Labor’s Bureau of Labor Statistics reports that approximately 1% of the US population is employed in the protective service occupation category, which includes police and correctional officers, security guards, and bailiffs [20]. Because these data do not include family members of individuals in law enforcement occupations, it is not known how our sample compares to the general public. Further research is warranted to determine if respondents’ exposure to law enforcement occupations biased their approval of police access.

Finally, survey respondents may have differed from the general population in relevant ways that our survey did not capture. For example, survey participants recruited by MTurk may spend more time online and therefore may be more comfortable with third party access to personal data. However, US respondents of other MTurk surveys reported spending similar periods of time online as the US general public [11] and being more (rather than less) concerned about their online privacy even after controlling for demographic factors, including age [21].

In light of these limitations, additional research is needed to assess whether our survey findings are generalizable. But even if they are, and it is shown that public support for law enforcement access to genetic genealogy databases is high, restrictions on police access could be desirable for reasons that are not yet widely appreciated. Forensic use of genetic genealogy databases makes potential suspects of large populations of individuals for the sole reason that they chose to participate in such databases. All of these individuals, except perhaps one, are actually innocent of the specific crime under investigation. But the odds are such that investigators sometimes target the wrong person. This occurred earlier in the hunt for the Golden State Killer, when investigators honed in on an elderly man living in an Oregon nursing home, whose daughter had uploaded her genetic data to a genealogy service called Ysearch.org [2].

Although the man was later cleared, such missteps can have enormous emotional and reputational impacts on those thrust unfairly into the investigative spotlight. While a suspect, an individual might be subjected to a range of invasive detective techniques, including personal surveillance, searches of financial records, and extensive interviews of family, friends, employers, and neighbors. Yet as noted by law professor Erin Murphy, “no law mandates that, once a name is formally cleared, the officer return and assure the suspect’s family and coworkers that he is truly as innocent as he was the day before the investigation began” [22]. Moreover, in some states, investigators may maintain the cleared suspect’s genetic information in informal databases not subject to federal oversight [4, 22]. Instances in which false leads were pursued as a result of matches to genetic genealogy databases were not described in the survey, which was conducted soon after DeAngelo was arrested, and are generally not well known.

Yet they could become more common as the universe of genetic services intended to facilitate personal discovery expands and more people participate in it. The DTC genetic genealogy testing market more than doubled in 2017, reaching over 12 million customers by year end [23]. All of the major players in this market, including AncestryDNA, 23andMe, and Family Tree DNA, provide customers the option of downloading their raw genetic data [24]. For some time, genetic research studies like Harvard’s Personal Genome Project and the University of Michigan’s Genes for Good have also given participants this option, and they have recently been joined by the National Institutes of Health’s *All of Us* Research Program, which will conduct genetic analyses for a subset of the 1 million anticipated participants [25, 26]. Some scholars have predicted that individual access to raw genetic data will soon “become expected or required as genomics becomes more clinically oriented and the public begins to insist on participatory data governance” [26].

Meanwhile, the kind and number of online services available to individuals in possession of their genetic data are growing. Some, including GEDmatch, help individuals understand their ancestry or identify genetic relatives. Others, like Promethease and DNA.Land, generate health, wellness, or trait reports interpreting personal genetic data; provide links to scientific literature or curated archives relevant to those data; or connect users to individuals with similar genetic variants or researchers studying them [24]. As the capabilities of these services continue to expand, it is likely the public will increasingly engage them, especially given that user satisfaction is generally high [27].

Although the landscape of personal genetic services is diverse and ever changing, a common practice among these services is to retain copies of the data that users share with them. Yet there is no guarantee that data shared by users actually belong to them. With the notable (and new) exception of GEDmatch [12], most personal genetic services forbid users from uploading data belonging to others, at least without authority to do so. But it is not clear how—or even if—these rules are being enforced. We may be comfortable requiring individuals who engage in the personal genetic landscape to accept the risk that law enforcement will search their data. But those whose genetic identities are being shared online without their knowledge are not aware that they are participating in this landscape and so cannot be said to have accepted its risks. When the police reach through participants to identify their relatives, those relatives also are unknowing and therefore nonconsenting. Future qualitative research should probe family members of genetic genealogy database participants to understand their perceptions of these privacy and other risks.

As more people become familiar with the vulnerabilities of personal genetic services, opinions may shift regarding the acceptability of police access to data that are generated by and shared with these services. In the meantime, however, policy discussions about whether to place limits on access should at least take into account the various purposes for which access may be sought, as some purposes may be more socially acceptable than others. While perceived invasions of privacy appear to be tolerable when the purpose is to catch violent or particularly depraved offenders, it seems that many would draw a line at searching their data to solve more ordinary crimes. Notably, this conclusion extends to communications providers with whom individuals share their social media and cell phone information, although it may be at odds with the US Supreme Court’s recent decision in *Carpenter versus US*, which held that individuals have a legitimate expectation of privacy in historical cell phone information that bars police from accessing those data without a warrant [13]. To the extent that police access to familial genetic data should depend on investigative purpose, however, the challenge is distinguishing those criminal circumstances that are sufficiently serious to justify access from those that are not [4, 16]. Yet another policy complexity concerns whether a court, commission, or other third party should be responsible for reviewing access requests to ensure that those distinctions, once made, are respected [22].

Far from being a forensic anomaly, the public genetic search that led to the arrest of the Golden State Killer suspect is quickly on its way to becoming routine procedure. What limits, if any, to place on police access to genetic genealogy databases must be thoughtfully considered and soon, with robust input from the public.

## Supporting information

S1 Text Methods and data analysis. (PDF)

S2 Text Survey. (PDF)

S1 Data Raw survey data. (XLSX)

## Abbreviations

DTC: direct-to-consumer
FBI: Federal Bureau of Investigation
