# Supplementary material for: Should police have access to genetic genealogy databases? Capturing the Golden State Killer and other criminals using a controversial new forensic technique
Source: PLoS Biol. 2018 Oct 2;16(10):e2006906. doi: 10.1371/journal.pbio.2006906 (PMC6168121; doi:10.1371/journal.pbio.2006906)
Supplement: S1 Text — (PDF) [file pbio.2006906.s001.pdf]

S1 Text. Methods & Data Analysis

Methods

We conducted the survey online in the United States in May 2018 using Amazon Mechanical Turk (MTurk). MTurk is an online marketplace used in crowdsourcing recruitment for survey research. The survey was constructed and administered on Qualtrics and a link to the survey was provided to qualified MTurk participants.

We restricted participation to individuals who were 18 years of age or older and located in the United States and paid them $0.25US for taking the survey. To control for data quality, we also restricted participation to individuals with high (≥92%) approval ratings for previous MTurk tasks and used attention check questions to identify inattentive participants and remove their responses from analysis. Respondents were restricted from taking the survey more than once.

All survey materials were approved by the Baylor College of Medicine Institutional Review Board (Protocol H37283). Participants were provided information about the nature of the survey and consent was obtained by clicking on the forward arrow to participate. The survey was found to be eligible for waiver of the requirement for written documentation of informed consent because the risks to participants were minimal and the study involved no procedures for which written consent is normally required outside of the research context.

Data Analysis

We calculated descriptive statistics including frequencies for respondents' characteristics and responses to survey questions. We explored differences between respondents' support of law enforcement's use of genetic information to identify perpetrators of violent crimes, non-violent crimes, crimes against children, and missing persons using McNemar's test, based on respondents' characteristics as appropriate. We also used McNemar's test to assess the differences between respondents' support of law enforcement's different activities, such as searching genealogic websites that match DNA to relatives and creating fake profiles of individuals on genealogical websites, based on respondents' characteristics as appropriate. We utilized McNemar's test in the aforementioned analyses because the independent variable(s) included paired, nominal data and the dependent variable(s) included dichotomous data.

Questions about the respondents' support of law enforcement's different activities and identifying purposes were measured nominally (dichotomous, Yes/No). Race and ethnicity were transformed into a dichotomous variable (non-Hispanic white vs. other), as presented in Table 1 of participant characteristics. Age and income were collected as ordinal variables and were treated as continuous variables in the regression model. Response options included for age: (coded as 0) 18-22, (1) 23-36, (2) 37-51, (3) 52-70, (4) 71-88, and (5) 89 or older. No respondents reported being 89 or older. Response options included for income: (coded as 0) <$10,000, (1) $10,000 - $19,999, (2) $20,000 - $34,999, (3) $35,000 - $49,999, (4) $50,000 - $74,999, (5) $75,000 - $99,999, (6) $100,000 - $149,999, and (7) >$150,000 .

Given the pattern of responses across purposes by activity, we conducted exploratory analyses to

assess whether there were underlying or latent factors within the activities. Using exploratory factor analysis with a varimax rotation (a type of orthogonal factor rotation), we found two latent factors that explained 48% of the variance with Eigenvalue factor loadings ranging from .604 - .813 for each factor. Items loading onto the first factor included all activities except for non-violent crimes which demonstrated good internal consistency ($\alpha = 0.907$), suggesting factor reliability. Factor scores (standardized to reflect z-scores) for the all but non-violent factor were then used as an outcome variable in a multivariate regression model to determine participant characteristics predictive of support. Gender and race/ethnicity were dummy coded for the regression model with male gender and non-Hispanic white at 0. The results indicated that the overall model was a significant predictor of support for the non-violent factor, $F(10,1567) = 3.373$, $p < 0.001$.

We conducted statistical analyses using SPSS 24 (IBM Corp., Armonk N.Y., USA). All *P* values were two-sided and with statistical significance set at $P < 0.05$.
