# Supplementary material for: Should police have access to genetic genealogy databases? Capturing the Golden State Killer and other criminals using a controversial new forensic technique
Source: PLoS Biol. 2018 Oct 2;16(10):e2006906. doi: 10.1371/journal.pbio.2006906 (PMC6168121; doi:10.1371/journal.pbio.2006906)
Supplement: S2 Text — (PDF) [file pbio.2006906.s002.pdf]

S2. Survey

Q1 We are inviting you to participate in a research study being conducted by Amy McGuire, JD, PhD, and Christi Guerrini, JD, MPH, in the Center for Medical Ethics and Health Policy at Baylor College of Medicine.

Participation in this study involves filling out an online survey about your views toward law enforcement use of genetic information to identify suspects. We will also ask you some questions about yourself, including your age and interactions with law enforcement. We will **not** ask you for any personally identifying information (such as your name or email address).

You must be at least 18 years old to take this survey.

This survey should take you less than 5 minutes to complete. Your participation is completely voluntary, and you may stop at any time for any reason.

If you are willing to participate, please click the forward arrow below.

Q2 **Genetic information** is the information contained in your genes. Genes are pieces of DNA that give your body the instructions it needs to develop and work. DNA stores this information in a code that you inherit from your parents and pass on to your children. It is possible to learn the identity of your blood relatives from your genetic information.

Genealogy is the study of family relatives. Some genealogical websites allow people to upload their genetic information to an online database that is searchable by other users. The practice helps people find family members because blood relatives share closely related genetic traits. Examples of genealogical websites are Ancestry.com and GEDmatch.

Individuals can obtain their genetic information from **direct-to-consumer genetic testing companies**. Customers provide saliva or blood samples to these companies, which then extract and process DNA from the samples. An example of a direct-to-consumer genetic testing company is 23andMe.

Q3 Recently, investigators used a genealogical website called GEDmatch to identify the alleged Golden State Killer, who killed a dozen individuals and raped at least 50 women throughout California in the 1970's and 1980's. First, investigators created a profile for the suspect on GEDmatch using a fake name and uploaded the suspect's DNA from samples left at crime scenes. The website then compared the suspect's genetic information to the genetic information of other users, and based on that comparison, identified a relative of the suspect. Finally, investigators matched the suspect's DNA to the DNA from the crime scene samples.

Q4 Should law enforcement be allowed to search genealogical websites that match DNA to relatives for the purpose of:

	Yes	No
Identifying perpetrators of violent crimes (for example: rape, murder, arson, kidnapping)	○	○
Identifying perpetrators of non-violent crimes (for example: car theft, drug possession)	○	○
Identifying perpetrators of crimes against children (for example: child abuse)	○	○
Identifying missing persons	○	○

Q5 Should law enforcement be allowed to create fake profiles of individuals on genealogical websites for the purpose of:

	Yes	No
Identifying perpetrators of violent crimes (for example: rape, murder, arson, kidnapping)	○	○
Identifying perpetrators of non-violent crimes (for example: car theft, drug possession)	○	○
Identifying perpetrators of crimes against children (for example: child abuse)	○	○
Identifying missing persons	○	○

Q6 Select the no choice below.

- ○ Yes
- ○ No

Q7 Should law enforcement be allowed to require direct-to-consumer genetic testing companies to reveal information about their customers (for example: name or form of payment) to law enforcement for the purpose of:

	Yes	No
Identifying perpetrators of violent crimes (for example: rape, murder, arson, kidnapping)	○	○
Identifying perpetrators of non-violent crimes (for example: car theft, drug possession)	○	○
Identifying perpetrators of crimes against children (for example: child abuse)	○	○
Identifying missing persons	○	○

Q8 Should law enforcement be allowed to search cell phone records for the purpose of:

	Yes	No
Identifying perpetrators of violent crimes (for example: rape, murder, arson, kidnapping)	○	○
Identifying perpetrators of non-violent crimes (for example: car theft, drug possession)	○	○
Identifying perpetrators of crimes against children (for example: child abuse)	○	○
Identifying missing persons	○	○

Q9 Should law enforcement be allowed to search social media accounts (for example: Facebook) for the purpose of:

	Yes	No
Identifying perpetrators of violent crimes (for example: rape, murder, arson, kidnapping)	○	○
Identifying perpetrators of non-violent crimes (for example: car theft, drug possession)	○	○
Identifying perpetrators of crimes against children (for example: child abuse)	○	○
Identifying missing persons	○	○

Q10 What is your age?

- ○ 18-22
- ○ 23-36
- ○ 37-51
- ○ 52-70
- ○ 71-88
- ○ 89 or older

Q11 How do you describe your gender?

- ○ Male
- ○ Female
- ○ Other (please specify): [text box]

Q12 Have you ever been arrested for a crime?

- ○ Yes
- ○ No

Q13 Have any of your family members ever been arrested for a crime?

- ○ Yes
- ○ No

Q14 Have you ever been convicted of a crime?

- ○ Yes
- ○ No

Q15 Please select the yes option below.

- ○ Yes
- ○ No

Q16 Have any of your family members ever been convicted of a crime?

- ○ Yes
- ○ No

Q17 Have you ever been the victim of a crime?

- ○ Yes
- ○ No

Q18 Have any of your family members ever been the victim of a crime?

- ○ Yes
- ○ No

Q19 Have you ever had a job in law enforcement? (for example: police officer, security guard, bailiff)

- ○ Yes
- ○ No

Q20 Have any of your family members ever had a job in law enforcement? (for example: police officer, security guard, bailiff)

- ○ Yes
- ○ No

Q21 Have you ever purchased products or services from a direct-to-consumer genetic testing company? (for example: 23andMe)

- ○ Yes
- ○ No

Q22 Have you ever researched relatives on a genealogical website? (for example: Ancestry.com)

- ○ Yes
- ○ No

Q23 What was your total household income (before taxes) from all sources in the last year?

- ○ Less than $10,000
- ○ $10,000 - $19,999
- ○ $20,000 - $34,999
- ○ $35,000 - $49,999
- ○ $50,000 - $74,999
- ○ $75,000 - $99,999
- ○ $100,000 - $149,999
- ○ $150,000 or more

Q24 Are you of Hispanic, Latino, or Spanish origin?

- ○ Yes
- ○ No

Q25 How do you describe your race? Please select all that apply.

- ○ American Indian or Alaska Native
- ○ Asian
- ○ Black or African American
- ○ Native Hawaiian or Other Pacific Islander
- ○ White
- ○ Other, please specify: [free text box]
